# Magnesium hydroxide versus macrogol/electrolytes in the prevention of opioid-induced constipation in incurable cancer patients: study protocol for an open-label, randomized controlled trial (the OMAMA study)

**DOI:** 10.1186/s12904-023-01143-2

**Published:** 2023-03-14

**Authors:** K.R.J. Kistemaker, A. de Graeff, M. Crul, G. de Klerk, P.M. van de Ven, M.P. van der Meulen, L. van Zuylen, M.A.H. Steegers

**Affiliations:** 1 Amsterdam UMC Location Vrije Universiteit Amsterdam, Medical Oncology, De Boelelaan 1117, Amsterdam, The Netherlands; 2 Amsterdam UMC Location Vrije Universiteit Amsterdam, Anesthesiology, De Boelelaan 1117, Amsterdam, The Netherlands; 3 Cancer Center Amsterdam, Treatment and Quality of Life, Amsterdam, The Netherlands; 4 Department of Medical Oncology, University Medical Center Utrecht, Utrecht, Academic Hospice Demeter, De Bilt, The Netherlands; 5 Amsterdam UMC Location Vrije Universiteit Amsterdam, Clinical Pharmacology and Pharmacy, De Boelelaan 1117, Amsterdam, The Netherlands; 6 Spaarne Gasthuis Location Hoofddorp, Medical Oncology, Spaarnepoort 1, Hoofddorp, The Netherlands; 7 Department of Data Science and Biostatistics, University Medical Center Utrecht, Utrecht, The Netherlands; 8 Department of Epidemiology and Health Economics, University Medical Center Utrecht, Utrecht, The Netherlands

**Keywords:** Opioids, Constipation, Magnesium hydroxide, Macrogol/electrolytes, Laxative, Cancer, Palliative care, Clinical trial

## Abstract

**Background:**

Opioid-induced constipation (OIC) is a common symptom in cancer patients treated with opioids with a prevalence of up to 59%. International guidelines recommend standard laxatives such as macrogol/electrolytes and magnesium hydroxide to prevent OIC, although evidence from randomized controlled trials is largely lacking. The aim of our study is to compare magnesium hydroxide with macrogol /electrolytes in the prevention of OIC in patients with incurable cancer and to compare side-effects, tolerability and cost-effectiveness.

**Methods:**

Our study is an open-label, randomized, multicenter study to examine if magnesium hydroxide is non-inferior to macrogol/electrolytes in the prevention of OIC. In total, 330 patients with incurable cancer, starting with opioids for pain management, will be randomized to treatment with either macrogol/electrolytes or magnesium hydroxide. The primary outcome measure is the proportion of patients with a score of < 30 on the Bowel Function Index (BFI), measured on day 14.

The Rome IV criteria for constipation, side effects of and satisfaction with laxatives, pain scores, quality of life (using the EQ-5D-5L), daily use of laxatives and escape medication, and cost-effectiveness will also be assessed.

**Discussion:**

In this study we aim to examine if magnesium hydroxide is non-inferior to macrogol/electrolytes in the prevention of OIC. The outcome of our study will contribute to prevention of OIC and scientific evidence of guidelines on (opioid-induced) constipation.

**Trial registration:**

This trial is registered at clinicaltrials.gov: NCT05216328 and in the Dutch trial register: NTR80508. EudraCT number 2022–000408-36.

## Background

Opioid-induced constipation (OIC) is a common symptom in cancer patients treated for pain and occurs in up to 59% [1]. It has a significant negative effect on the quality of life of patients, due to physical problems, psychological problems and social consequences [1–5]. OIC may cause problems that range from daily discomfort with social insecurity and disability to intestinal obstruction. This leads to limitations in self-management and a risk of need for more care, including hospital admission. Constipation is even more burdensome in the palliative phase of cancer, when opioid use is most common due to a high prevalence of pain and other risk factors for constipation [1, 6]. Thus, attention for prevention and early recognition of OIC is needed.

Laxatives are recommended for prevention of OIC, although there is little evidence to support this [7]. One randomized, open-label study compared macrogol/electrolytes with sodium picosulphate and lactulose in ambulatory cancer patients starting with opioids and showed that both macrogol/electrolytes and sodium picosulphate were more effective than lactulose in preventing OIC [8].

Another laxative used for prevention of OIC is magnesium hydroxide, a magnesium compound also used as an antacid. The use of magnesium compounds for constipation dates back to as early as the eighth century [9]. Over the years different kinds of magnesium compounds (e.g. magnesium oxide, magnesium hydroxide, magnesium citrate) have been used [9]. As magnesium oxide is transformed in the stomach to magnesium hydroxide, these drugs are considered to be the same. Nowadays, magnesium hydroxide is the most commonly used magnesium compound in the United States and Europe [9]. There has been only one randomized study about prevention of OIC by a magnesium compound. This recent study compared magnesium oxide with naldemedine (a peripheral μ-receptor antagonist) in preventing OIC. It showed that naldemedine resulted in significantly better constipation-related quality of life, measured with two different questionnaires, better stool consistency and less constipation based on Rome IV criteria, both after two weeks and three months of treatment [10]. There were no statistical differences in number of spontaneous bowel movements per week and in quality of life as measured with the short form-36.

At present, naldemedine is not yet globally available [11] and costs on average 376,74 dollars (348,19 euros) for a 30-day supply [12], making it is less accessible compared to traditional and widely available laxatives such as macrogol/electrolytes or magnesium hydroxide with a cost of around 5 euros (5,40 dollars) for a 30-day supply [13, 14].

Despite the lack of evidence from other randomized controlled trials on the prevention of OIC by the more traditional laxatives, Dutch and other national guidelines on constipation in cancer patients recommend either macrogol/electrolytes or magnesium compounds (including magnesium hydroxide) as first-line laxative treatment to prevent OIC [15–17].

To gain evidence for these guideline recommendations, the OMAMA study examines if magnesium hydroxide is non-inferior to macrogol/electrolytes in the prevention of OIC in patients with metastatic or locally advanced incurable cancer and compares side-effects, tolerability and cost-effectiveness.

## Methods

### Study design

The OMAMA study is an open-label, randomized, multi-center study to examine whether magnesium hydroxide is non-inferior to macrogol/electrolytes in the prevention of OIC. This study will be conducted in eleven hospitals throughout The Netherlands. The trial is designed in accordance with the Declaration of Helsinki and the principles of Good Clinical Practice (GCP) set by the International Conference of Harmonization (ICH) [18, 19]. It has been approved by the Medical research Ethics Committee of Amsterdam UMC. The study is registered in the Dutch Trial Register, identifier 80508; EudraCT number 2022–000408-36. Patient recruitment and data collection have started in October 2022.

### Study population

Both patients from the outpatient clinic and hospitalized patients from eleven academic and non-academic hospitals will be recruited. We will ask patients with metastatic or locally advanced incurable cancer to participate in this study at the moment that they will start to use slow release or transdermal opioids for pain. Additional inclusion criteria are that the patient is ≥ 18 years old and able to complete a Dutch questionnaire. Previous treatment with opioids is allowed, if discontinued for more than four weeks before the start of the study.

Exclusion criteria for participation include patients with contra-indications for laxatives; use of laxatives during the previous four weeks; severely impaired renal function, defined as a serum creatinine > 180 umol/l; and an estimated life expectancy < 3 months.

### Informed consent procedure

Patients will be asked to participate in the study by the physician who prescribes the opioid for pain and will give them a package containing the patient information leaflet (PIL) and the informed consent form. In case of a patient in the outpatient clinic, the patient will be given the time to read the PIL directly after the consult. The local investigator will then give additional information, answer all questions and ask for informed consent. In case of a hospitalized patient, the patient will receive the PIL and informed consent form on the day that opioid use will start. The local investigator will then meet the patient the next day to give additional information, answer all questions and ask for informed consent. When informed consent is given, the PIL is signed twice by the patient and the investigator. After signing informed consent patients are free to withdraw from the trial at any moment.

### Randomization, blinding and treatment allocation

Patients will be randomized after fulfilling eligibility criteria and written informed consent. The randomization will be 1:1 using computerized block randomization with varying block sizes of 2 and 4 in random order. Randomization will be stratified by center and presence of constipation at the start of the study, defined as a score ≥ 30 on the Bowel Function Index (BFI, see paragraph ‘study measurements’). Randomization will be performed by the local researcher after baseline assessments.

### Treatment

Patients will be treated with either macrogol/electrolytes or magnesium hydroxide in a standard dosing regimen. Macrogol/electrolytes is started at a dose of 1 sachet (containing 13,125 g of macrogol) once daily and magnesium hydroxide at a dose of 724 mg t.i.d., both orally. Macrogol/electrolytes is regarded as the standard treatment (usual care) to which magnesium hydroxide will be compared. The dose of macrogol/electrolytes and magnesium hydroxide may be increased to 2 sachets daily or 1448 mg t.i.d., respectively, or may be decreased or discontinued because of intolerance or side effects during the study period. To determine whether a change of the dosage is needed, a contact moment with the local investigator by telephone will take place on day 7.

### Study measurements

Demographic and baseline data will be extracted from the medical record. This includes sex, age, performance status, comorbidities, treatment with systemic and/or radiotherapy during the study period and the type and dose (converted to morphine equivalents) of the opioid which the patient will use.

The primary outcome measure is the proportion of patients with a score of < 30 on the Bowel Function Index (BFI), measured on day 14. The BFI is a clinician-administered, patient-reported questionnaire to assess clinically significant constipation, validated in patients receiving opioids for chronic non-malignant and malignant pain [20–22]. The BFI consists of three questions, assessing ease of defecation, feeling of incomplete bowel evacuation and personal judgement of the patient regarding constipation, each during the last 7 days and each rated on a scale of 0 (best possible outcome) to 100 (worst possible outcome). A total score ≥ 30 (mean of the three separate scores) indicates clinically significant constipation. A change of the total score of > 12 is regarded as clinically meaningful. The BFI has been used in a large ‘real world’ observational study in cancer patients receiving opioids [1] and in randomized trials of opioid antagonists for OIC [23–26]. It has been recommended as the assessment tool of choice for OIC [7, 27]. The BFI will be administered by the local investigator on day 0 (at the start of the study, after giving informed consent and before the randomization) and by telephone on day 14.

As a secondary outcome measure, the local investigator will assess the Rome IV criteria for opioid-induced constipation on day 0 and by telephone on day 14 (see Table 1). At least two of the six criteria have to be fulfilled in order to diagnose constipation.

Another secondary outcome measure is the 5-level EuroQol-5 Dimension questionnaire (EQ-5D-5L) completed on day 0 and 14. The EQ-5D-5L is a self-administered, generic assessment tool developed by the EuroQol Group to assess the quality of life and to determine cost-effectiveness. This measurement tool consists of five questions on dimensions of health (i.e., mobility, self-care, pain/discomfort, usual activities, and anxiety/depression) [28].

Other secondary outcome measures include a pain score (Numeric Rating Scale, ranging from 0 to 10, on days 0 and 14), a questionnaire about patient satisfaction with the laxative on day 14 (one question on a four-point Likert scale, ranging from unsatisfied to very satisfied), a questionnaire about side effects of the laxatives on day 14 and a questionnaire about medical consumption (the modified iMTA Medical Consumption Questionnaire (iMCQ)) on day 14 [29]. An overview of the measurement tools can be found in Table 1.

Lastly, patients will report daily laxative use in a medication diary to evaluate protocol adherence and also daily use of escape medication.

**Table 1:** Overview of measurement tools

Measurement tool	
Bowel Function Index (Day 0 and day 14)	3 questions about defecation, rated on a scale of 0 (best possible outcome) to 100 (worst possible outcome). Total score ≥ 30 (mean of the three separate scores) indicates clinically significant constipation. A change of the total score of > 12 is regarded as clinically meaningful.
Rome IV criteria for opioid-induced constipation (Day 0 and day 14)	At least two of the following criteria have to be fulfilled in order to diagnose constipation: - Straining > 25% of defecations - Lumpy or hard stools in > 25% of defecations - Sensation of incomplete evacuation in > 25% of defecations - Sensation of anorectal obstruction/blockage in > 25% of defecations - Manual manoeuvres to facilitate > 25% of defecations - < 3 spontaneous defecations per week
EQ-5D-5L questionnaire (Day 0 and day 14)	5 questions on dimension of health: mobility, self-care, pain/discomfort, usual activities, and anxiety/depression. Each dimension consists of 5 levels: no problems, slight problems, moderate problems, severe problems and extreme problems
Pain (Day 0 and day 14)	Numeric rating scale (0 = no pain, 10 = worst pain imaginable) Satisfaction with laxatives will be scored on a four-point Likert scale, ranging from unsatisfied to very satisfied Side effects of laxatives (i.e. bad taste, flatulence and nausea) will be scored on a four-point Likert scale (not at all, a bit, rather, very much)
Patient satisfaction (Day 14)	
Side effects (Day 14)	
Modified iMCQ (Day 14)	9 questions related to frequent occurring contact with healthcare providers. Each answer is dichotomous (yes/no) with the option to add the number of visits if the answer ‘yes’ is filled in.

### Sample size calculation

The sample size was determined to achieve 90% power to declare non-inferiority of magnesium hydroxide in case the proportion of patients with a score < 30 on the BFI at day 14 to be equal for magnesium hydroxide and macrogol/electrolytes assuming one-sided testing at a significance level of 2.5% [30]. The sample size was based on two non-randomized studies that showed that approximately 40% of patients starting with opioids and not using laxatives do not develop constipation after 14 days [31, 32]. Prophylaxis with laxatives can only be effective in (part of) the remaining 60% of patients. Treatment with macrogol/elektrolytes is effective in 70% of patients with symptomatic chronic constipation [33, 34]. Based on these data, we estimated the percentage of patients without constipation (BFI < 30) after 14 days of treatment with macrogol/electrolytes to be 82% (40% plus 0,7 × 60%). The main considerations are that the proportion of patients with a BFI < 30 under magnesium hydroxide at the non-inferiority margin should clearly be higher than the estimated 40% of patients without opioid induced constipation and the clinical judgement that more than 15% difference could no longer be translated in a conclusion that magnesium hydroxide is non inferior to macrogol/electrolytes. Based on the previous data, we calculated a sample size of 138 evaluable patients per arm. We will increase the inclusion with approximately 20% to anticipate potential loss to follow-up to N = 165 per arm.

### Statistical analysis

All data will be coded and collected in a secure cloud-based clinical data management platform (Castor EDC). Analysis will be performed using the latest version of IBM SPSS Statistics.

Primary analysis for non-inferiority will be performed both on a protocol and intention-to-treat basis. The primary outcome is the difference in proportion of responders (BFI < 30 at day 14) between the two arms. The primary analysis will use the Newcombe score method to estimate a one-sided 97.5% confidence interval for the difference in proportion of responders (proportion responders under magnesium hydroxide minus proportion responder under macrogol/electrolytes). The null hypothesis (H0: Magnesium hydroxide is inferior to macrogol/electrolytes in the prevention of OIC) will be rejected and non-inferiority will be claimed for magnesium hydroxide if the lower boundary of the 97.5% confidence interval is larger than the prespecified non-inferiority margin of -15%. An additional analysis will be performed in which the risk difference for response is estimated using a generalized linear model for a binomial outcome and an identity link, while adjusting for the stratification factors of center and constipation at the start of the study.

An additional per protocol analysis will be performed using inverse-probability-of-treatment-weighing (IPTW) in the intention-to-treat population. For this IPTW analysis, logistic regression analysis in the intention-to-treat population will be used to predict the probability of a subject being adherent based on predefined confounders. In this analysis, non-adherence is defined as < 80% of the prescribed laxative being taken as determined from the patient diary [35]. The inverse of the predicted probabilities will then be used as weights in an analysis comparing treatment outcomes between the groups and to estimate a per-protocol effect. In this weighted analysis, the risk difference for response and its two sided 95% confidence interval is estimated using generalized linear models for a binomial outcome and an identity link, while adjusting for the stratification factors.

Secondary analyses include the same analyses for constipation judged by professional care givers, based on the Rome IV criteria for opioid-induced constipation. Furthermore, we aim to study baseline predictors of the response to macrogol/electrolytes and magnesium hydroxide in patients starting with opioids, including sex, age, performance status and comorbidity.

Lastly, the cost-effectiveness of macrogol/electrolyte compared to magnesium hydroxide will also be a secondary outcome. The cost-effectiveness will primarily be influenced by the effect of the medication on healthcare resource use and quality of life, since their unit costs are similar. In this analysis, we will compare the cost-effectiveness of macrogol/electrolyte and magnesium hydroxide in patients. The analysis will be performed from a healthcare perspective with a time horizon of two weeks. A decision tree will be developed with branches for initial therapy and responders and non-responders. The costs and quality of life of the patients in the trial will be used as input for this decision tree. This requires data collection on healthcare procedures and quality of life in responders, non-responders and patients with side-effects of laxatives for the follow-up period of the study (two weeks). These healthcare procedures will be extracted from the modified iMCQ after two weeks. The modified iMCQ will be adjusted in such a way that resource use due to gastrointestinal complaints will only be measured in these patients with intensive healthcare resource use. All individual health care procedures will be linked to their unit costs. Reimbursement prices issued by the Dutch Healthcare Authority (NZa) and national reference prices will be used for this assessment as outlined in current Dutch pharmaco-economic guidance. Quality of life will be measured using the EQ-5D-5L at baseline and after two weeks.

A scenario will be added to the analyses for the use of naloxegol and naldemedine as a first-line treatment in case of OIC. Naloxegol and naldemedine are peripheral µ-receptor antagonists that have shown to be effective for the treatment of OIC [36]. There are no studies available yet evaluating the efficacy of naloxegol for prevention of OIC. For naldemedine there is one recent randomized controlled trial available that has evaluated the efficacy of naldemedine for prevention of OIC [10]. As no effect data of first-line treatment with naloxegol or naldemedine will be measured during the trial, the scenario analysis will be an early health-economic analysis. In this analysis we will use effect data based on literature and expert opinion and will perform a threshold analysis to estimate the effect size needed to result in a cost-effective intervention. Bootstrapping will be performed to assess the uncertainty of costs and utility loss of (sub)groups, while deterministic (one-way) and probabilistic sensitivity analyses will be performed to assess the uncertainty of the analyses in the decision tree.

With regards to missing data, we do not plan imputation for the main outcome. If unexpectedly outcomes are missing, we will perform sensitivity analyses using single-value imputation under the most extremes scenarios. Multiple imputation may be considered if important confounders are missing or if missing on the main outcome is higher than currently anticipated.

## Discussion

The OMAMA study is an open-label, randomized controlled trial that examines if magnesium hydroxide is non-inferior to macrogol /electrolytes in the prevention of opioid-induced constipation (OIC) in patients with metastatic or locally advanced incurable cancer. OIC is a symptom that often occurs in palliative care and can have a negative impact on the quality of life of patients. In current practice both macrogol/electrolytes and magnesium hydroxide are prescribed for the prevention of OIC. Yet, scientific evidence in the form of randomized controlled trials is largely lacking. The OMAMA study will provide evidence on the prevention of OIC, the tolerability and side-effects of both laxatives.

Research in palliative patients can be challenging and has been controversial for a long time. Often the ethical concern is whether it is morally justified to let these patients participate in research [37, 38]. They are considered vulnerable and fragile due to their illness and/or multiple co-morbidities with a high risk of drop-out [39]. However, multiple studies showed that palliative patients are willing to participate in medical research and may benefit from their participation [37, 39, 40]. Nonetheless, it is important to minimize the burden when conducting research with this group of patients [41]. In our study the extra burden for the patient will be minor. If the patient does not participate in the study, the physician will also prescribe either macrogol/electrolytes or magnesium hydroxide (depending on the treating physician or the protocol of the hospital) to prevent constipation. Furthermore, the study period is only fourteen days during which patients will have to complete the questionnaires only at the beginning and at the end of the study period. During the process of writing the study protocol and standard operating procedures we have asked two cancer patients for comments and for help to minimise the burden for the patient.

There is relatively little time given to the patient for the informed consent. Patients in the outpatients clinic will be asked for informed consent after the consult with their physician and hospitalized patients will be asked informed consent within 24 h after starting with opioids. However, waiting longer with starting a laxative may be detrimental to the patient. Moreover, our study is a low-intervention trial and as stated above, the burden for the patient will be minor.

Our study period of fourteen days is based on previous studies which show that it is sufficient time to evaluate the development of constipation [10, 31, 32]. Two prospective non-randomized studies compared the incidence of OIC after 14 days with or without prophylactic laxatives and found an incidence of 48% [31] and 34% [32] in the group treated with prophylactic laxatives versus an incidence of 65% [31] and 55% [32] in the group treated without prophylactic laxatives, respectively. They concluded that prophylactic laxatives were associated with a lower incidence of OIC. These data suggest that approximately 60% of patients starting with opioids and not using laxatives will have developed constipation after 14 days and 40% will not. Moreover, studies have shown that a single administration of opioids can already cause constipation [42–44]. Consequently, after 14 days of therapeutic opioid doses it will be possible to evaluate if a patient has developed constipation or not.

In this study we have not included a placebo treatment group, because the aforementioned studies already have shown that approximately 60 percent of the patient starting with opioids will develop constipation when no laxative is prescribed [31, 32]. Therefore, a placebo arm was thought to be unethical.

Our study has some limitations. First of all, our study focuses on a specific patient group (i.e. opioid-naive patients with incurable cancer) and the question may arise whether the results can be extrapolated to non-cancer patients. However, cancer patients might have OIC that is more difficult to treat than OIC in non-cancer patients, due to additional factors contributing to constipation. These factors include tumor growth in the gastrointestinal tract, decreased mobility, inadequate oral intake of fluids and dietary fibers and use of other constipation-inducing drugs such as antiemetics and anticholinergic drugs [45, 46]. Moreover, most cancer patients are elderly, who already have reduced colonic motility due to changes caused by aging [47]. Hence, if prevention of OIC with either laxative is successful in these cancer patients, it will probably also be successful in non-cancer patients.

Another limitation is that the EQ-5D-5L that is used in this study is a general tool to measure the quality of life. There exists a more specific, but much more extensive tool to measure quality of life related to constipation, the Patient Assessment of Constipation Quality of Life (PAC-QOL) [48]. The PAC-QOL consists of 28 questions. Since the patients already have to complete various other questionnaires, it was thought that adding this questionnaire would increase the burden on the patient and potentially increase the chance of missing data. Furthermore, the EQ-5D is a commonly used tool in cost-effectiveness analyses [49].

## Conclusion

The OMAMA study will evaluate if magnesium hydroxide is non-inferior to macrogol/electrolytes in the prevention of opioid-induced constipation in patients with metastatic or locally advanced incurable cancer. Both laxatives are commonly used as a preventive medicine, yet scientific evidence is scarce. Our study will provide evidence on the prevention of OIC and will compare the tolerability and side-effects of both laxatives. The OMAMA study has started recruitment in October 2022 and is expected to be completed in 2 years.

## Data Availability

Not applicable.

## Abbreviations

OIC: Opioid-induced constipation
GCP: Good Clinical Practice
ICH: International Conference of Harmonization
PIL: Patient information leaflet
BFI: Bowel Function Index
EQ-5D-5L: 5-Level EuroQol-5 Dimension
iMCQ: iMTA Medical Consumption Questionnaire
EMA: European Medicines Agency
IPTW: Inverse-probability-of-treatment-weighting
NZa: Nederlandse Zorgautoriteit (Dutch Healthcare Authority)
PAC-QOL: Patient Assessment of Constipation Quality of Life

## References

[1] Davies A, Leach C, Butler C, Gregory A, Henshaw S, Minton O (2021). Opioid-induced constipation in patients with cancer: a "real-world," multicentre, observational study of diagnostic criteria and clinical features. Pain.

[2] Bell T, Annunziata K, Leslie JB (2009). Opioid-induced constipation negatively impacts pain management, productivity, and health-related quality of life: findings from the National Health and Wellness Survey. J Opioid Manag.

[3] Christensen HN, Olsson U, From J, Breivik H (2016). Opioid-induced constipation, use of laxatives, and health-related quality of life. Scand J Pain.

[4] Dhingra L, Shuk E, Grossman B, Strada A, Wald E, Portenoy A (2013). A qualitative study to explore psychological distress and illness burden associated with opioid-induced constipation in cancer patients with advanced disease. Palliat Med.

[5] Penning-van Beest FJ, van den Haak P, Klok RM, Prevoo YF, van der Peet DL, Herings RM (2010). Quality of life in relation to constipation among opioid users. J Med Econ.

[6] Snijders RAH, Brom L, Theunissen M, Van den Beuken -van Everdingen MHJ (2023). Update on Prevalence of Pain in Patients with Cancer 2022: A Systematic Literature Review and Meta-Analysis. Cancers.

[7] Farmer AD, Drewes AM, Chiarioni G, De Giorgio R, O'Brien T, Morlion B (2019). Pathophysiology and management of opioid-induced constipation: European expert consensus statement. United European Gastroenterol J.

[8] Wirz S, Nadstawek J, Elsen C, Junker U, Wartenberg HC (2012). Laxative management in ambulatory cancer patients on opioid therapy: a prospective, open-label investigation of polyethylene glycol, sodium picosulphate and lactulose. Eur J Cancer Care (Engl).

[9] Mori H, Tack J, Suzuki H (2021). Magnesium Oxide in Constipation. Nutrients..

[10] Ozaki A, Kessoku T, Tanaka K, Yamamoto A, Takahashi K, Takeda Y (2022). Effectiveness of Naldemedine Compared with Magnesium Oxide in Preventing Opioid-Induced Constipation: A Randomized Controlled Trial. Cancers (Basel)..

[11] Blair HA (2019). Naldemedine: A Review in Opioid-Induced Constipation. Drugs.

[12] Hu K, Bridgeman MB (2018). Naldemedine (Symproic) for the Treatment Of Opioid-Induced Constipation. P T.

[13] National Healthcare Institute. Available from: https://www.medicijnkosten.nl/medicijn?artikel=MACROGOL+EN+ELECTR+TEVA+POEDER+V+DRANK+IN+SACHET&id=a4b9021a035a42763895573d45078263. Accessed 19 Jan 2023.

[14] National Healthcare Institute. Available from: https://www.medicijnkosten.nl/medicijn?artikel=MAGNESIUMHYDROXIDE+TEVA+KAUWTABLET+724MG&id=d12def31c492a5b5c30599b30bc3f383. Accessed 20 Jan 2023.

[15] Larkin PJ, Cherny NI, La Carpia D, Guglielmo M, Ostgathe C, Scotte F (2018). Diagnosis, assessment and management of constipation in advanced cancer: ESMO Clinical Practice Guidelines. Ann Oncol..

[16] Crockett SD, Greer KB, Heidelbaugh JJ, Falck-Ytter Y, Hanson BJ, Sultan S (2019). American Gastroenterological Association Institute Guideline on the Medical Management of Opioid-Induced Constipation. Gastroenterology.

[17] Palliaweb. Guideline Constipation in the palliative phase. Available from: https://palliaweb.nl/richtlijnen-palliatieve-zorg/richtlijn/obstipatie/preventie/opioidgeinduceerde-obsitapatie. Accessed 16 Nov 2022.

[18] World Medical Association (2013). Declaration of Helsinki: ethical principles for medical research involving human subjects. JAMA.

[19] ICH. Integrated addendum to ICH E6(R1): guideline for good clinical practice E6(R2) Current Step 4 version. 2016;2:1–60. Available from: https://database.ich.org/sites/default/files/E6_R2_Addendum.pdf. Accessed 6 Nov 2022.

[20] Abramowitz L, Beziaud N, Causse C, Chuberre B, Allaert FA, Perrot S (2013). Further validation of the psychometric properties of the Bowel Function Index for evaluating opioid-induced constipation (OIC). J Med Econ.

[21] Rentz AM, van Hanswijck de Jonge P, Leyendecker P, Hopp M (2011). Observational, nonintervention, multicenter study for validation of the Bowel Function Index for constipation in European countries. Curr Med Res Opin..

[22] Rentz AM, Yu R, Muller-Lissner S, Leyendecker P (2009). Validation of the Bowel Function Index to detect clinically meaningful changes in opioid-induced constipation. J Med Econ.

[23] Ahmedzai SH, Nauck F, Bar-Sela G, Bosse B, Leyendecker P, Hopp M (2012). A randomized, double-blind, active-controlled, double-dummy, parallel-group study to determine the safety and efficacy of oxycodone/naloxone prolonged-release tablets in patients with moderate/severe, chronic cancer pain. Palliat Med.

[24] Dupoiron D, Stachowiak A, Loewenstein O, Ellery A, Kremers W, Bosse B (2017). A phase III randomized controlled study on the efficacy and improved bowel function of prolonged-release (PR) oxycodone-naloxone (up to 160/80 mg daily) vs oxycodone PR. Eur J Pain.

[25] Leng X, Zhang F, Yao S, Weng X, Lu K, Chen G (2020). Prolonged-Release (PR) Oxycodone/Naloxone Improves Bowel Function Compared with Oxycodone PR and Provides Effective Analgesia in Chinese Patients with Non-malignant Pain: A Randomized. Double-Blind Trial Adv Ther.

[26] Poelaert J, Koopmans-Klein G, Dioh A, Louis F, Gorissen M, Loge D (2015). Treatment with prolonged-release oxycodone/naloxone improves pain relief and opioid-induced constipation compared with prolonged-release oxycodone in patients with chronic severe pain and laxative-refractory constipation. Clin Ther.

[27] Argoff CE, Brennan MJ, Camilleri M, Davies A, Fudin J, Galluzzi KE (2015). Consensus Recommendations on Initiating Prescription Therapies for Opioid-Induced Constipation. Pain Med.

[28] Pickard AS, Wilke CT, Lin HW, Lloyd A (2007). Health utilities using the EQ-5D in studies of cancer. Pharmacoeconomics.

[29] iMTA Productivity and Health Research Group. Manual iMTA Medical Cost Questionnaire (iMCQ) Rotterdam: iMTA. Erasmus University Rotterdam; 2018. Available from: https://www.imta.nl/assets/uploads/2022/01/EN-iMCQ-questionnaire-2018-SAMPLE.pdf. Accessed 2 Nov 2022.

[30] Head SJ, Kaul S, Bogers AJ, Kappetein AP (2012). Non-inferiority study design: lessons to be learned from cardiovascular trials. Eur Heart J.

[31] Tokoro A, Imai H, Fumita S, Harada T, Noriyuki T, Gamoh M (2019). Incidence of opioid-induced constipation in Japanese patients with cancer pain: A prospective observational cohort study. Cancer Med.

[32] Ishihara M, Ikesue H, Matsunaga H, Suemaru K, Kitaichi K, Suetsugu K (2012). A multi-institutional study analyzing effect of prophylactic medication for prevention of opioid-induced gastrointestinal dysfunction. Clin J Pain.

[33] Lyseng-Williamson KA (2018). Macrogol (polyethylene glycol) 4000 without electrolytes in the symptomatic treatment of chronic constipation: a profile of its use. Drugs Ther Perspect.

[34] Taylor RR, Guest JF (2010). The cost-effectiveness of macrogol 3350 compared to lactulose in the treatment of adults suffering from chronic constipation in the UK. Aliment Pharmacol Ther.

[35] Karve S, Cleves MA, Helm M, Hudson TJ, West DS, Martin BC (2009). Good and poor adherence: optimal cut-point for adherence measures using administrative claims data. Curr Med Res Opin.

[36] Rekatsina M, Paladini A, Drewes AM, Ayob F, Viswanath O, Urits I (2021). Efficacy and Safety of Peripherally Acting mu-Opioid Receptor Antagonist (PAMORAs) for the Management of Patients With Opioid-Induced Constipation: A Systematic Review. Cureus.

[37] Gysels M, Evans CJ, Lewis P, Speck P, Benalia H, Preston NJ (2013). MORECare research methods guidance development: recommendations for ethical issues in palliative and end-of-life care research. Palliat Med.

[38] Kars MC, van Thiel GJ, van der Graaf R, Moors M, de Graeff A, van Delden JJ (2016). A systematic review of reasons for gatekeeping in palliative care research. Palliat Med.

[39] White C, Hardy J (2010). What do palliative care patients and their relatives think about research in palliative care?-a systematic review. Support Care Cancer.

[40] Gysels MH, Evans C, Higginson IJ (2012). Patient, caregiver, health professional and researcher views and experiences of participating in research at the end of life: a critical interpretive synthesis of the literature. BMC Med Res Methodol.

[41] DeCamp M, Alasmar A, Fischer S, Kutner JS (2022). Meeting ethical challenges with authenticity when engaging patients and families in end-of-life and palliative care research: a qualitative study. BMC Palliat Care.

[42] Dorn S, Lembo A, Cremonini F (2014). Opioid-induced bowel dysfunction: epidemiology, pathophysiology, diagnosis, and initial therapeutic approach. Am J Gastroenterol Suppl.

[43] Ketwaroo GA, Cheng V, Lembo A (2013). Opioid-induced bowel dysfunction. Curr Gastroenterol Rep.

[44] Mehendale SR, Yuan CS (2006). Opioid-induced gastrointestinal dysfunction. Dig Dis.

[45] Clemens KE, Klaschik E (2008). Management of constipation in palliative care patients. Curr Opin Support Palliat Care.

[46] Varrassi G, Banerji V, Gianni W, Marinangeli F, Pinto C (2021). Impact and Consequences of Opioid-Induced Constipation: A Survey of Patients. Pain Ther.

[47] Rumman A, Gallinger ZR, Liu LWC (2016). Opioid induced constipation in cancer patients: pathophysiology, diagnosis and treatment. Expert Rev Qual Life Cancer Care.

[48] Marquis P, De La Loge C, Dubois D, McDermott A, Chassany O (2005). Development and validation of the Patient Assessment of Constipation Quality of Life questionnaire. Scand J Gastroenterol.

[49] Kharroubi SA, Edlin R, Meads D, Browne C, Brown J, McCabe C (2015). Use of Bayesian Markov chain Monte Carlo methods to estimate EQ-5D utility scores from EORTC QLQ data in myeloma for use in cost-effectiveness analysis. Med Decis Making.

